# Is the vagus nerve our neural connectome?

**DOI:** 10.7554/eLife.35592

**Published:** 2018-03-16

**Authors:** V Reggie Edgerton, Parag Gad

**Affiliations:** 1 Department of Integrative Biology and PhysiologyUniversity of California, Los AngelesLos AngelesUnited States

**Keywords:** spinal cord injury, physical rehabilitation, vagus nerve stimulation, neural plasticity, Rat

## Abstract

What are the implications of the vagus nerve being able to mediate the time-dependent plasticity of an array of sensorimotor networks?

**Related research article** Ganzer PD, Darrow MJ, Meyers EC, Solorzano BR, Ruiz AD, Robertson NM, Adcock KS, James JT, Jeong HS, Becker AM, Goldberg MP, Pruitt DT, Hays SA, Kilgard MP, Rennaker RL. 2018. Closed-loop neuromodulation restores network connectivity and motor control after spinal cord injury. *eLife* **7**:e32058. doi: 10.7554/eLife.32058

The vagus nerve reports on the state of many of the organs in our body, including the heart, the lungs and the gut, and it relays this information to various neural control networks that unconsciously regulate internal organs. It has also been shown that artificial electric stimulation of the vagus nerve helps with recovery in animal models of stroke, tinnitus and spinal cord injury (De Ridder et al., 2014; Hays, 2016). In particular, stimulation of the vagus nerve promotes the recuperation of motor skills and, maybe, autonomic functions (such as breathing), even when the injuries took place years before the intervention. However, we do not fully understand how stimulating this single nerve can lead to such results.

Now, in eLife, Patrick Ganzer, Robert Rennaker at the University of Texas at Dallas and the Texas Biomedical Device Center, and colleagues, report that stimulating the vagus nerve of a rat with spinal injuries helps it to recover mobility of an affected limb – in this case, its front paw (Ganzer et al., 2018). The stimulation has to be applied during a short time window after the rat manages to perform a specific movement with this paw, such as grasping a lever with a specific level of strength.

In this scenario, grasping the lever activates a network of neurons. The connections between these neurons will then be reinforced if the vagus nerve is stimulated within seconds of this task being completed (Figure 1). Classical long-term potentiation experiments show that simultaneous activation leaves ‘tags’ in neurons, which help to strengthen any connections between these neurons (He et al., 2015). Yet, during vagus nerve stimulation, there is no direct synaptic link between the circuits that perform the motor task and the synapses that are excited by the vagus nerve (Alvarez-Dieppa et al., 2016; Hulsey et al., 2017).

**Figure 1.**
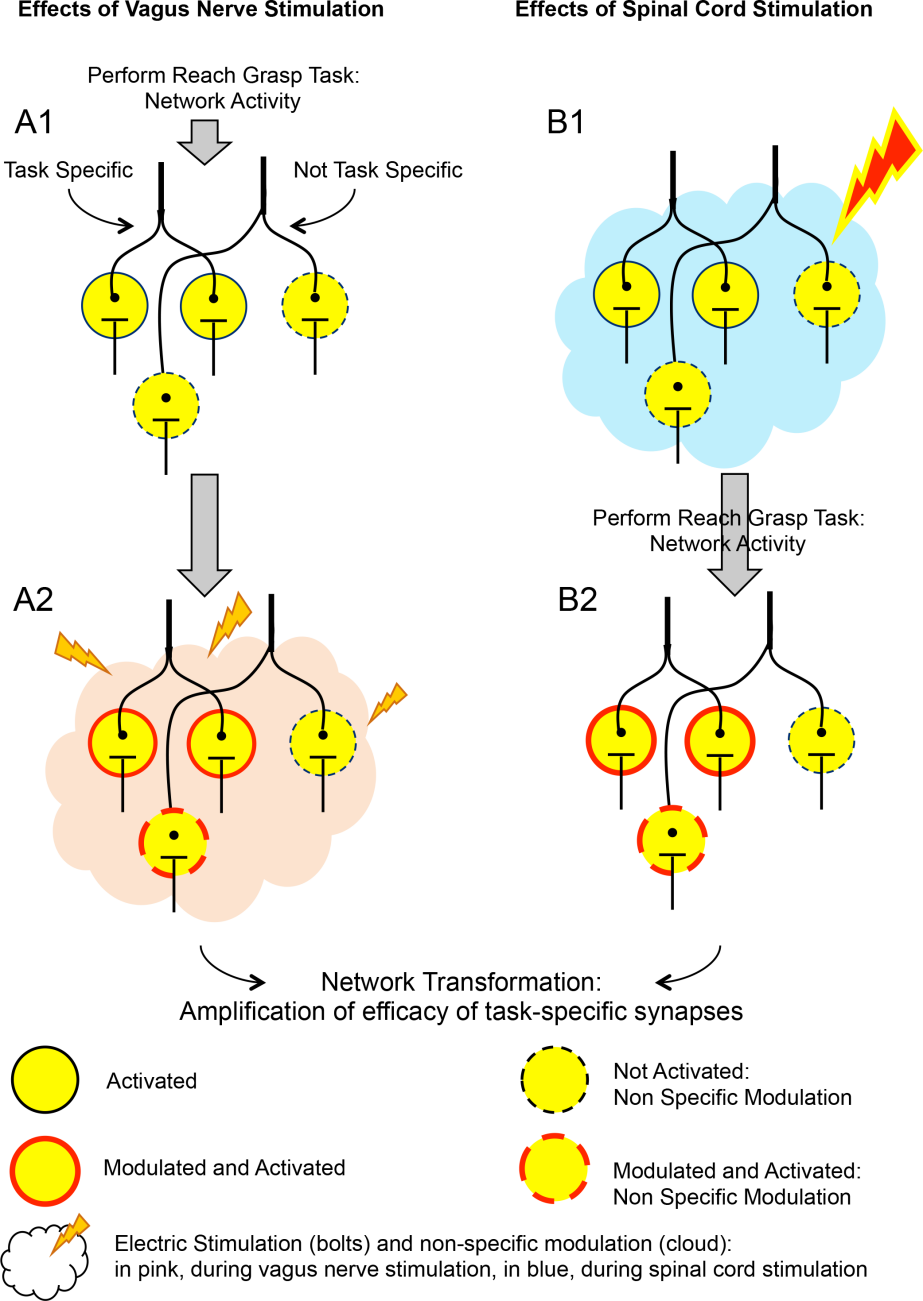
The effects of vagus nerve and spinal cord stimulations on neural networks. The schematic shows the effect of two types of artificial stimulations on a network of neurons (circles), which is activated when a rat with spinal cord injury pulls a lever with its affected limb. Column A shows the effect of vagus nerve stimulation on neural networks that are activated when the rat pulls that lever. In A1, when the rat performs the task, it activates neurons. Some of these are specific to this task (encircled by solid black lines), some of which are not (dashed black line). In A2, the vagus nerve (not represented) is stimulated (pink cloud and lightning bolts). The neurons previously activated are also modulated by this stimulation, and certain new neurons are activated (dashed red line). This combination of activation and modulation strengthens the connections between the neurons that previously fired together, which leads to a transformation of the network. Ultimately, this more robust network supports better physical performance by the rats. Column B shows the effect of spinal cord stimulation on the same neural networks. In B1, spinal cord stimulation is applied (blue cloud and lighting bolt) before the task takes place. When the task is performed (B2), the synapses between the groups of neurons that are involved are further reinforced, a result that is comparable to what is obtained with vagus nerve stimulation. Similar results may also be obtained by performing the stimulation during the task itself. Both vagus nerve and spinal cord stimulations depend on the subject performing a certain task that activates the networks which need reinforcing. However, how these two types of stimulations differ, and how they modulate neural connections, remains unclear.

IMAGE CREDITS: Figure courtesy of V Reggie Edgerton.

Instead, the effects of vagus nerve stimulation could be mediated by a wide range of neuromodulatory mechanisms, which are non-specific to a given synapse. Because the vagus nerve is connected to so many different organs, both in sensorimotor and autonomic ways, it provides the entry point to various neuroendocrine and neurotransmitter systems. Essentially, it seems that the vagus nerve can form a 'connectome' for many functions, which means that interventions via the vagus nerve have the potential to help with the recovery of multiple functions.

Remarkably, similar effects can be obtained by stimulating the spinal cord before the action is performed (Figure 1; Gad et al., 2013). In both cases, there are activity-dependent mechanisms that identify the synapses that have been activated, and the stimulation triggers a series of time-dependent events that reinforce the connections between the relevant neurons.

More broadly, there are at least four fundamental biological concepts relevant to the findings by Ganzer et al. First, the vagus nerve mediates physiological systems (including sensorimotor and autonomic systems) which are extensively and comprehensively integrated together. Second, these systems are dynamic and can reorganize and interact in ways that can dramatically change our behavior. Third, time-dependence has a central role in defining activity-dependent plasticity. Fourth, the networks under the influence of the vagus nerve may be constantly reshaped by neural and biochemical signals via activity-dependent mechanisms.

These four points are consistent with the theory of neural group selection put forward by Gerald Edelman, the American biologist who shared the Nobel Prize in Physiology or Medicine in 1972 (Edelman, 1987). According to this theory, during early and neonatal development neurons form somewhat malleable connections based on genetic (i.e., internal) signals. Thereafter, throughout life, these networks dynamically and continuously create different combinations of functional neuronal connections – called neural networks, or somatically formed groups – by functionally pruning or reinforcing the strength of their connections. This remodeling is determined by the level of activity of the neural connections as they are constantly responding to internal and external stimuli. Finally, which neural groups fire together determines the overall shape of these networks. In terms of behavior, the basic functional unit of the brain is therefore not a neuron, but instead a group of connections among neurons that tend to fire together. Thus, fully understanding the mechanisms at play during vagus nerve stimulation requires thinking at the level of systems, as well as of subcellular components.
